# Progress in Surgery

**Published:** 1903-01-10

**Authors:** 


### PROGRESS IN SURGERY.

(*Concluded from page* 233.)

### OTOLOGY.

**Infective Lateral Sinus Thrombosis.**—T. M Callender[5] records an interesting case of this kind successfully treated. The patient, a man of 38, complained first of a foul ear-discharge and general weakness, but not of pain in the head. The day after admission to the hospital the patient had a violent rigor, and the temperature ran up to 105·8°. A quantity of foul pus was evacuated by opening up the mastoid. The temperature rose again and a rigor occurred two days after the operation. Then stiffness of the neck developed, but without pain or tenderness. The lateral sinus was exposed and a hypodermic needle passed into it, through which a little sero-pus escaped. The jugular vein was ligatured in two places and divided, and the lateral sinus freely opened. A catheter was passed into the upper end of the vein, and through it the lateral sinus was irrigated from below up, and a large quantity of very foul pus and clotted blood was thus removed. The vein was ligatured above the hole made for the catheter, and the wound in the neck closed and the lateral sinus packed. The patient's recovery was complicated with the development of a large pyæmic abscess in the back, fœtid bronchitis, and an abscess in the base of the right lung. A pneumothorax developed after an attempt to aspirate the lung abscess, and from that time the lung condition improved. The neck wound suppurated. The facial nerve was unfortunately damaged. Charles A. Ballance[6] has discussed the question of ligaturing the jugular vein in cases of aural pyæmia. Much divergence of opinion exists in reference to this question both in England and abroad, but as the writer observes, "when once the pathology of an affection is clearly appreciated divergence of view as to its treatment ought to disappear." In the development of pyæmia or septicæmia from ear disease, thrombosis of the sinus is really an accidental phenomenon, not an essential one, and is a protective measure which is successful if the clot remains uninfected. It is the secondary infection of the clot and not the occurrence of thrombosis which constitutes the danger. The writer has had a case in which pyæmia was due to infective thrombosis of the superior petrosal sinus without thrombosis of the lateral sinus. If when removing septic material from the lateral sinus we restrict ourselves to removing pus and broken-down clot, and leave apparently sound clot untouched, we may overlook a spreading "disconnected" thrombus on the far side of the sound portion. In these cases, the author concludes, the first thing to be determined is whether the patient is suffering from an acute systemic affection, or from a systemic disturbance depending upon a local process. If this cannot be determined beforehand, it must be determined as early as possible during the operation on the temporal bone. The operation on the vein for its occlusion should be undertaken (*a*) in acute pyæmia or septicæmia whether clot is present or not; (*b*) if the sinus wall is gangrenous or its contents are putrefying, unless it is perfectly clear that on both sides of the area of inflammation the lumen of the sinus is completely blocked by non-infected thrombi—a condition seldom realised; (*c*) if it is proved, or even suspected, that the blood of the jugular bulb is in part or wholly clotted; and (*d*) if the jugular vein is thrombosed. The vein should be tied immediately after the decision to tie it has been arrived at, without previously beginning or completing the operation on the sinus region. Ablation of the vein up to the bulb is better than ligature. If the vein is thrombosed its ablation will begin at the junction with the subclavian, if not it may begin about the level of the cricoid cartilage. The operation on the sinus should be completed by removing bone so as to expose the sinus towards the torcular to a distance at least three-quarters of an inch beyond the area of inflammatory change, and downwards probably it is best to remove bone as far as the jugular bulb.

**Otitic Brain Abscess.**—W. P. Eagleton (Newark, U.S.A.),[7] has read a paper on this subject. Literature showed that chronic suppuration of the ear was responsible for 84 per cent., and acute suppuration for the remaining 16 per cent. of the cases. Cerebellar abscess was apt to be multiple, which fact explained many failures in treatment. Many of the cases of abscess were chronic, remaining quiescent for months or years. Pain in the head might be the only symptom. In one case, for a long time pain in a molar tooth was the only symptom, and after operation the subsequent dressings produced similar pain in the tooth. Aphasia was the only reliable localising symptom. The operation was usually exploratory in nature, which fact should be remembered. As an illustration of this it had been found that in about 10 per cent. of cases operated upon the symptoms caused abscess to be suspected to exist in the side opposite to that on which it really existed. In 21 per cent. of 101 cases operated on, the abscess was not found, either because the pus was too thick to pass along the aspirating needle, or because the capsule of the abscess was unusually thick and resisting. It was therefore justifiable to pass the finger into the brain if the abscess could not be otherwise detected. A large opening for drainage should be made. R. H. Woods[8] records a successful case of operation for otitic temporo-sphenoidal abscess. The symptoms included fœtid ear discharge, slow cerebration, irregular temperature, double optic neuritis, sluggish pupils, and some degree of amnesic aphasia. There was no paralysis. At the first operation the mastoid antrum was drained, fœtid pus escaping, the lateral sinus scraped, and pus evacuated from an extradural abscess in the cerebellar fossa. Two days later a second operation was performed, as the symptoms still persisted. A hollow needle was first passed three times into the cerebellum with negative results. Then the needle was similarly inserted into the temporo-sphenoidal lobe, and on the third insertion pus was found. A crucial incision was then made in the dura mater, and forceps passed along the needle track, and 4 drachms of horribly fœtid pus and brain sloughs evacuated. The cavity was washed out with weak carbolic and a drainage-tube inserted. The patient completely recovered, the only permanent effect left behind was that his mental condition was such that he was more "huffy," more inclined to think himself injured on the slightest provocation, than before. D. B. Lees [9] has placed on record two cases of temporo-sphenoidal brain abscess in which no suppurative disease of the middle ear was present. At the post-mortems on these cases necrosis was found of a small portion of the petrous bone over the auditory meatus in one and over the tympanum in the other, but the mastoid cells and middle ears were healthy in each. It seemed as if the bone had been the primary seat of disease. The cases prove that such brain abscesses may exist without otorrhœa and with the tympanic membrane normal.

[5] Lancet, Sept. 20, 1902, p. 812. [6] Lancet, Ibid., p. 795. [7] Med Rec., Aug. 20, 1902 p. 194. [8] Journ. L. R. and O., July, 1902, p 341. [9] Vide Abst. Journ. L. R. and O., Ibid., p. 391.

